# Management of Drug-Induced Psoriasis With Secukinumab in a Patient With Schizoaffective Disorder: A Case Report

**DOI:** 10.7759/cureus.65229

**Published:** 2024-07-23

**Authors:** Ibraheim Ayub, Shehzad Ayub

**Affiliations:** 1 Internal Medicine, Yale New Haven Hospital, Waterbury, USA; 2 Psychiatry, University of Arizona College of Medicine - Phoenix, Phoenix, USA

**Keywords:** psoriasis, suicidal ideations, psychodermatology, schizoaffective disorder, lithium, secukinumab, drug-induced psoriasis

## Abstract

Psoriasis is a chronic inflammatory systemic disorder often associated with psychiatric comorbidities such as depression and anxiety. This case report describes a 25-year-old man with schizoaffective disorder, bipolar type, whose suicidal ideation was worsened by a severe psoriasis flare that was induced by the initiation of lithium for psychiatric management. Lithium was switched to valproate, and treatment with secukinumab, an IL-17A inhibitor, was initiated, resulting in significant improvement in both psoriasis and mental health. This case highlights the phenomenon of drug-induced psoriasis, emphasizing the need for a high index of suspicion and careful review of past medical history. It underscores the reciprocal relationship between psoriasis and psychiatric comorbidities, advocating for a tailored approach to treating both conditions to achieve optimal outcomes.

## Introduction

Psoriasis is a chronic, systemic, inflammatory autoimmune disorder that primarily affects the skin and significantly impacts psychosocial health, affecting 125 million people globally [1]. Its pathogenesis involves a complex interaction between the immune system and epidermal keratinocytes, leading to the release of cytokines like tumor necrosis factor (TNF-α), interleukin (IL)-1β, IL-6, and IL-22 [2]. This causes inflammation and abnormal keratinocyte proliferation, resulting in erythematous, scaly plaques [2].

Recent studies reveal that 20% of adults with psoriasis suffer from depression, 21% from anxiety, and 0.77% experience suicidal thoughts [1]. In a case-control study, 84% of psoriasis patients were found to have psychiatric comorbidities, and further research underscored a more pronounced association between psoriasis and mental health disorders than with other dermatological conditions [3,4]. Moreover, these conditions significantly raise the risk of suicide [1,5]. The link between psoriasis and psychiatric comorbidities is complex, involving overlapping cytokine and neuropeptide pathways [4].

Recognizing the impact of psoriasis on mental health is crucial, as is treating the underlying mental health conditions. However, certain psychotropic medications can exacerbate or even cause psoriasis, further worsening the patient's mental health. Drug-induced psoriasis occurs when medications such as beta-blockers, lithium, hydroxychloroquine, and chloroquine either trigger de novo psoriasis or worsen existing psoriasis [6]. Lithium, commonly used for mood stabilization in bipolar disorder, affects secondary messenger systems, such as adenylyl cyclase and inositol monophosphate pathways, which are implicated in psoriasis exacerbation [7]. Additionally, some antidepressants like fluoxetine and bupropion, antipsychotics like olanzapine, quetiapine, and aripiprazole, as well as benzodiazepines, have been reported to worsen psoriasis [8-11].

Distinguishing between psoriasis and drug-induced psoriasis can be challenging both clinically and histopathologically; therefore, maintaining a high index of suspicion is important [12]. Treatment typically involves discontinuing the offending medication if possible and increasing the use of appropriate treatments to control the disease [12]. Treatments for psoriasis are classified into several categories, including topical treatments, such as corticosteroids and vitamin D analogs, phototherapy such as UVB phototherapy, systemic treatments, such as methotrexate and cyclosporine, and biologic agents such as TNF-alpha inhibitors and IL-17 inhibitors [6].

Individuals living with psoriasis often face social stigma that negatively impacts their quality of life [2]. Therefore, effective management of psoriasis, including appropriate mental health treatment, can significantly enhance self-esteem and overall well-being, as depicted in the case report.

## Case presentation

The patient is a 25-year-old male with a history of schizoaffective disorder, bipolar type, and a 15-year history of psoriasis. He was admitted to the psychiatric unit after presenting to the emergency department with suicidal ideation and a plan to cut his throat. Over the past month, his mood had been declining, significantly worsened by a severe flare-up of his psoriasis, which he described as the worst he had ever experienced. He endorsed neurovegetative symptoms of depression but denied current symptoms of mania or psychosis. The patient had been started on prednisone, ciprofloxacin, and trimethoprim-sulfamethoxazole due to concerns about a skin infection 1.5 weeks prior to this hospitalization. His psychotropic regimen included lithium 900 mg daily, escitalopram 10 mg daily, and olanzapine 15 mg daily. Lithium had been initiated a few months ago for mood stabilization.

During his stay in the psychiatric ward, the patient reported worsening mood and pain due to his extensive psoriasis characterized by a psoriasis area and severity index (PASI) of 42.3. Physical examination revealed widespread erythematous plaques with micaceous scale on his trunk, extremities, face, and scalp, covering approximately 80% of his body surface area. He developed new pustular lesions on the soles of his feet during his stay. His sleep quality was poor due to generalized pain from his psoriatic lesions and low back pain, which was attributed to psoriatic arthritis. His lithium levels at presentation were non-toxic at 0.5 mmol/L. The patient had previously undergone biologic therapy for his psoriasis, albeit with intermittent adherence, including trials of adalimumab and etanercept, with his last dose of adalimumab administered six months ago.

The severity of his psoriasis led to significant isolation, pain, and discomfort, exacerbating his depression and suicidal ideation. Given the impact of his psoriasis on his mood and psychosocial well-being, dermatology was consulted. After evaluating his psoriasis and considering the possibility of lithium-induced psoriasis, a decision was made to discontinue lithium, which had been started a few months prior and was suspected to exacerbate his condition. He was transitioned from lithium to valproate 1000 mg daily for mood stabilization. Valproate serum levels and liver function tests were monitored. No biopsy was performed, as the diagnosis of psoriasis was evident from clinical appearance and history. Ciprofloxacin and trimethoprim-sulfamethoxazole were discontinued, as there were no signs of active skin infection. Prednisone was discontinued in favor of triamcinolone cream and secukinumab. The patient received two doses of 150 mg of secukinumab subcutaneously, with a plan to repeat this weekly for five weeks, following the protocol for initiating secukinumab. During the hospitalization, he received three weeks’ worth of injections. The treatment regimen is detailed in Table 1.

**Table 1:** Treatment regimen

Medication	Dosage	Frequency	Route	Duration
Secukinumab	300mg	Weekly for five weeks, then every four weeks	Subcutaneous	Ongoing
Olanzapine	15mg	Nightly	Oral	Ongoing
Valproate	1000mg	Daily	Oral	Ongoing
Escitalopram	10mg	Daily	Oral	Ongoing
Triamcinolone acetonide	0.10%	Twice a Day	Topical	Two weeks, followed by a two-week break
Discontinued Medication	Dosage	Frequency	Route	Duration
Lithium	900mg	Daily	Oral	Discontinued
Prednisone	10mg	Daily	Oral	Discontinued
Ciprofloxacin	500mg	Twice a Day	Oral	Discontinued
Sulfamethoxazole-Trimethoprim	400mg-80mg	Twice a Day	Oral	Discontinued

**Figure 1:**
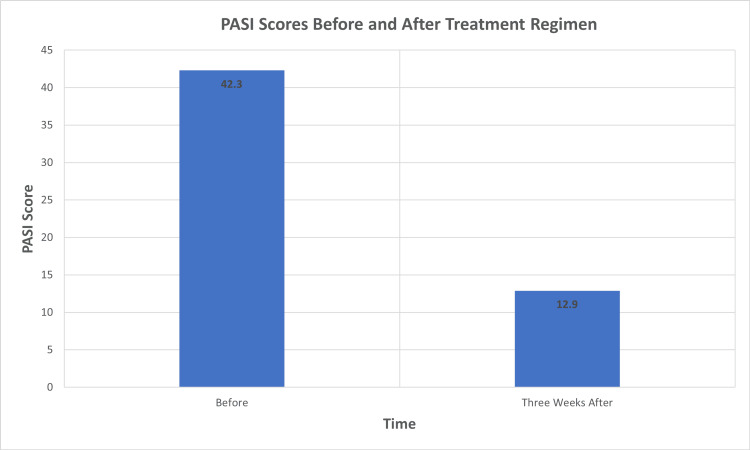
Comparison of PASI scores before and three weeks of initiating secukinumab and the treatment regimen PASI: psoriasis area and severity index

One week after initiating secukinumab, there was a 50% improvement in erythema, induration, and scale. Interestingly, two oval patches of skin, where he received the injections, were clear of psoriasis. After the second week, there was a dramatic improvement in his mood, depression, anxiety, and sleep quality. The patient became more social, interactive, and less isolated. Valproate levels were within the therapeutic range at 53.4 µg/mL. His psoriasis continued to improve after receiving his third weekly dose, with marked improvement in his mental status. He tolerated his medications well and experienced no adverse effects from discontinuing lithium. His PASI score after three weeks of treatment improved to 12.9 (Figure 1).

The patient was discharged with his new medication regimen, including ongoing treatment with secukinumab 300 mg once a week for two more weeks before transitioning to 300 mg every four weeks, as recommended.

## Discussion

This case report provides insight into the complex relationship between psychotropic medications and the exacerbation of psoriasis, emphasizing the need for careful management of patients with psoriasis and comorbid psychiatric conditions. It highlights the interplay between mental and physical health, stressing the importance of addressing both concurrently.

The patient experienced a severe and extensive exacerbation of psoriasis, covering approximately 80% of his body surface area with a PASI score of 42.3. This severe presentation significantly impacted the patient's quality of life, leading to social isolation, pain, and discomfort. Notably, the exacerbation precipitated a major depressive episode with suicidal ideation, necessitating psychiatric hospitalization. The development of new pustular lesions on the soles during hospitalization indicated the complexity of the condition, possibly representing palmoplantar pustular psoriasis, adding considerable discomfort and emphasizing the severity of this adverse event.

Patients with psoriasis exhibit higher rates of anxiety and depression compared to the general population [1]. The visible skin lesions contribute to social stigma, low self-esteem, and psychological distress [2]. While it is easy to attribute the increased rates of depression to social stigma, research suggests that psoriasis and depression may share common inflammatory pathways, predisposing individuals to both conditions [2]. High levels of pro-inflammatory cytokines, such as IL-6 and TNF-α, are shared by psoriasis and depression, leading to alterations in serotonin, norepinephrine, and dopamine metabolism in the limbic system and basal ganglia, contributing to depressive symptoms [13]. These same cytokines initiate the maturation of naïve T cells into Th17 helper cells, which play a role in psoriasis [13]. Additionally, depression leads to higher levels of Substance P, which increases the severity of pruritis, keratinocyte proliferation, and activation of lymphocytes, which all aggravate psoriasis [13]. In this case, the patient’s severe psoriasis exacerbation was closely linked to his deteriorating mood and increased suicidal ideation. This bidirectional relationship underscores the importance of addressing both dermatologic and psychiatric symptoms concurrently. Effective management of psoriasis can lead to substantial improvements in mental health, as observed in this patient’s case.

Lithium can induce or exacerbate psoriasis in patients with or without a prior history of the condition [6,7]. Manifestations can range from mild to severe, involving the face, scalp, trunk, and extremities, with plaque-type psoriasis being the most prevalent presentation [7]. The pathogenesis appears dose-related, potentially activating the inflammatory cascade and altering secondary messenger systems, which impact calcium homeostasis [7]. The decrease in cyclic adenosine monophosphate and inositol resulting from lithium treatment leads to low intracellular calcium levels, causing a lack of differentiation and increased proliferation of keratinocytes, as well as enhanced chemotaxis and phagocytic activity of neutrophils [7]. The onset of lithium-induced psoriasis varies, typically developing over several months, although exacerbations of pre-existing psoriasis may occur more rapidly [6]. While precise prevalence rates of psoriasis related to lithium are not well-documented, studies indicate a higher incidence in males [14].

The temporal association between the initiation of lithium therapy and the exacerbation of psoriasis in this case is noteworthy. The patient's psoriasis worsened significantly a few months after starting lithium, which aligns with the typical onset of lithium-induced psoriasis reported in the literature [6]. This temporal relationship, coupled with the dramatic improvement observed after discontinuing lithium and initiating alternative treatments, strongly suggests a causal link between lithium use and psoriasis exacerbation.

Clinicians must maintain a high index of suspicion and carefully review past medical history, medications, and family history to diagnose or prevent drug-induced psoriasis [12]. A biopsy is of limited utility in diagnosing drug-induced psoriasis, as it is mostly indistinguishable from psoriasis on histopathology [6]. Some pathological differences, such as a low number of Munro microabscesses, the presence of macrophages, and occasionally, an eosinophilic infiltrate, can be seen [6]. Additionally, drug-induced psoriasis often lacks tortuous papillary dermal capillaries. However, the absence of these changes does not preclude the diagnosis [6].

In this case, the patient’s worsening psoriasis, likely due to initiating lithium months prior, led to a psychosocial decline. Moderate to severe psoriasis should be considered a relative contraindication for lithium therapy [14]. This could have been prevented by avoiding medications known to exacerbate psoriasis, particularly given his history of moderate psoriasis and questionable adherence to treatment. Although prednisone is known to induce psychiatric symptoms, the patient reported a significant mood deterioration before its initiation. The patient attributed this decline in part to the exacerbation of his psoriasis, which he perceived as a contributing factor to his depression. Effective management of such cases requires a personalized approach to balance psychiatric stabilization and dermatological health.

The decision to transition from lithium to valproate was driven by the need to mitigate the severe exacerbation of psoriasis while maintaining mood stabilization. Valproate, another mood stabilizer, does not carry the same risk of worsening psoriasis, making it a suitable alternative in this scenario. The patient’s positive response to valproate, alongside the discontinuation of lithium, highlights the importance of personalized treatment plans. If the exacerbation had been milder, a placebo-controlled trial demonstrated that administering six grams of inositol daily could mitigate the severity of psoriatic lesions in patients undergoing lithium treatment [15]. Additionally, another case report has shown that treating lithium-exacerbated psoriasis with an appropriate biologic therapy while maintaining lithium can yield successful results [16].

In this case, we initiated secukinumab, a biologic therapy targeting interleukin-17A. Biologic therapies have revolutionized the treatment of psoriasis by targeting the specific underlying inflammatory pathways involved in the disease process. After the patient started on secukinumab, we observed significant improvement in his psoriasis, sleep, and mental health, eventually leading to his discharge from the hospital. His PASI improved from 42.3 to 12.9. This improvement underscores the efficacy of secukinumab in treating psoriasis and its potential to positively impact the patient’s psychosocial well-being.

## Conclusions

This case illustrates the complex interplay between severe psoriasis and psychiatric conditions, particularly how the psoriasis flare-up led to isolation, pain, discomfort, and exacerbation of psychosocial symptoms. The multidisciplinary approach to managing his condition, including the successful transition from lithium to valproate and the initiation of secukinumab, was crucial in improving the patient’s overall well-being and stabilizing his psychiatric and dermatological health.
